# Nearsighted

**Published:** 1881-12

**Authors:** 


### NEAR-SIGHTED.

Persons living in cities begin to wear glasses earlier than country people, from the want of opportunities of looking at things at a distance. Those who wish to put far off the evil day of "*spectacles*," should accustom themselves to long views. The eye is always relieved, and sees better, if, after reading a while, we direct the sight to some far-distant object, even for a minute. Great travellers and hunters are seldom near-sighted. Sailors discern objects at a great distance with considerable distinctness, when a common eye sees nothing at all. One is reported to have such an acute sight, that he could tell when he was going to see an object. On one occasion, when the ship was in a sinking condition, and all were exceedingly anxious for a sight of land, he reported from the look-out that he could not exactly see the shore, but he could pretty near do it.

